# Supplementary material for: Mutations in PCYT2 disrupt etherlipid biosynthesis and cause a complex hereditary spastic paraplegia
Source: Brain. 2019 Oct 22;142(11):3382–97. doi: 10.1093/brain/awz291 (PMC6821184; doi:10.1093/brain/awz291)
Supplement: awz291_Supplementary_Data [file awz291_supplementary_data.zip › awz291-Suppl_data/Supplementary_Data4.zip!/Supplementary information 3 - fibroblast lipidomics.zip!/Fibroblast lipidomics/Controle vs Patient1/Statistical plots and data/LPA species/Boxplots.pdf]

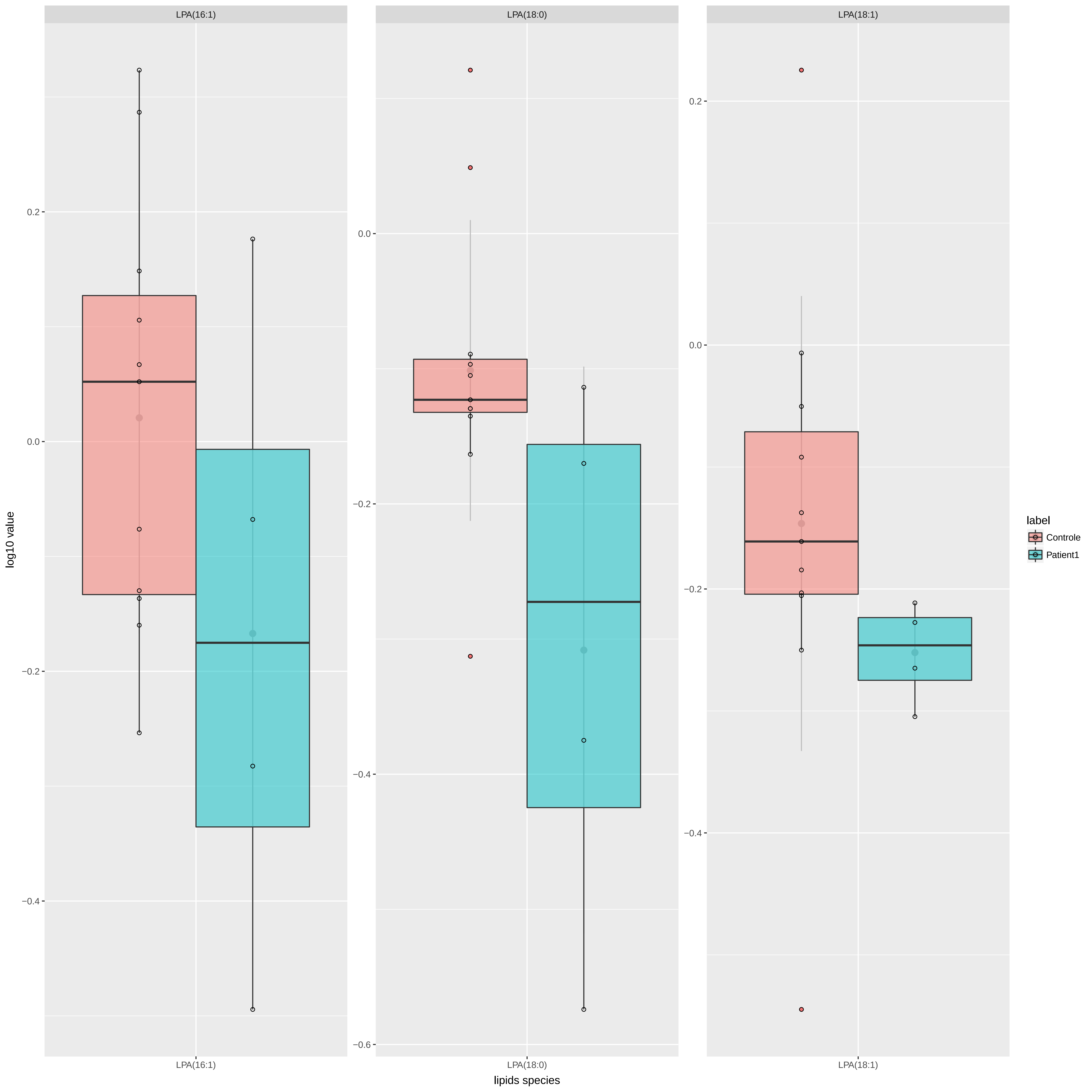

LPA(16:1)
LPA(18:0)
LPA(18:1)
0.2
0.0
−0.2
−0.4
0.0
−0.2
−0.4
−0.6
0.2
0.0
−0.2
−0.4
log10 value
lipids species
LPA(16:1)
LPA(18:0)
LPA(18:1)
label
Controle
Patient1
